# Oral health-related quality of life among adults undergoing orthodontic procedure with ceramic braces versus aligners

**DOI:** 10.6026/973206300211128

**Published:** 2025-05-31

**Authors:** Nidhi Singh, Shaivi Sharma, Shruti R. Varshney, Deepankar Bhatnagar, Geetika Tomer, Prachi Sethy, Pallavi Mishra, Aseem Sharma

**Affiliations:** 1 Department of Dentistry, Government Medical College, Pali, Rajasthan, India; 2 Department of Dentistry, Amaltas Medical College, Dewas, Madhya Pradesh, India; 3 Department of Orthodontics and Dentofacial Orthodpedics, KD Dental College, Mathura, Uttar Pradesh, India; 4 Department of Orthodontics and Dentofacial Orthopedics, MM College of Dental Sciences and Research, MMU, Ambala, Haryana, India; 5 Intern, Kalinga Institute of Dental Sciences, Kalinga Institute of Industrial Technology (KIIT), Deemed to be University, Patia, Bhubaneswar, Odisha, India; 6 Department of Oral and Maxillofacial Pathology, Kalinga Institute of Dental Sciences, Kalinga Institute of Industrial Technology (KIIT), Deemed to be University, Patia, Bhubaneswar, Odisha, India; 7 Department of Orthodontics and Dentofacial Orthopedics, HIDS Paonta Sahib, Himachal Pradesh, India

**Keywords:** Adult orthodontic treatment, aligners, ceramic braces, malocclusion, oral health-related quality of life (OHRQoL), orthodontics

## Abstract

The oral health-related quality of life in adults undergoing orthodontic treatment with ceramic braces versus aligners is of interest. A total of 200 adult patients were recruited, with 100 patients each undergoing treatment with ceramic braces or aligners. Both ceramic braces and aligners positively affect OHRQoL. However, aligners generally provide a more comfortable and aesthetically pleasing experience for adult patients.

## Background:

Orthodontic treatment is a cornerstone in modern dentistry, aimed at enhancing the appearance, alignment and functionality of teeth. Among the most common orthodontic appliances are ceramic braces and aligners, both of which offer distinct advantages depending on patient preferences and clinical requirements [1, 2]. Ceramic braces, known for their aesthetic appeal due to their tooth-colored brackets, are often used for their durability and effective treatment of complex malocclusions [3, 4]. On the other hand, aligners, particularly clear plastic trays like Invisalign, have gained popularity for their virtually invisible appearance, comfort and convenience [5, 6]. While these treatments can effectively address malocclusions and improve dental aesthetics, the impact on patients' oral health-related quality of life (OHRQoL) is a subject that has not been comprehensively studied, especially in adult populations [7, 8]. Oral health-related quality of life (OHRQoL) is a multidimensional construct that evaluates the impact of oral health on a person's daily life, including aspects such as functional limitations (*e.g.*, difficulty in eating, speaking), discomfort, social interactions and psychological well-being [9, 10-11]. Traditional studies on orthodontic treatment have predominantly focused on clinical outcomes like treatment duration, alignment improvements, or relapse rates [12, 13]. However, understanding the psychosocial and functional impacts of orthodontic treatments on patients is equally important for delivering holistic and patient-centred care [14]. Specifically, how treatment modalities such as ceramic braces or aligners affect patients' perceptions of their dental aesthetics, comfort and overall well-being remains underexplored [15]. In adults, the choice of orthodontic appliance can be influenced by several factors, such as professional appearance, lifestyle and treatment goals [16]. Therefore, it is of interest to assess the oral health-related quality of life among adults undergoing orthodontic procedure with ceramic braces versus aligners.

## Materials and Methods:

## Study design:

This was a prospective, observational, cross-sectional study conducted to evaluate the impact of orthodontic treatment with ceramic braces and aligners on the Oral Health-Related Quality of Life (OHRQoL) in adult patients. The study was designed to assess OHRQoL parameters such as aesthetics, discomfort, functional limitations and psychological impact and compare the two treatment modalities. The study was done after obtaining approval from institutional ethics committee and informed consent from the participants.

## Sample size:

A total of 200 adult patients were recruited for the study, with 100 patients undergoing orthodontic treatment with ceramic braces and 100 patients undergoing treatment with aligners. The sample size was determined using a power calculation to ensure sufficient statistical power to detect differences between the two groups.

## Inclusion criteria:

[1] Adults aged 18-40 years.

[2] Patients undergoing orthodontic treatment with either ceramic braces or aligners.

[3] Minimum treatment duration of 6 months to ensure that patients had adequate time to experience the full impact of their orthodontic treatment.

[4] Patients with mild to moderate malocclusions (Class I, Class II, or Class III) requiring orthodontic treatment.

## Exclusion criteria:

[1] Individuals with systemic diseases (*e.g.*, diabetes, hypertension) that could affect oral health or the ability to complete treatment.

[2] Patients with psychological disorders or cognitive impairments that could affect their responses to questionnaires.

[3] Patients who had previously undergone orthodontic treatment or had received prior dental interventions that might influence their OHRQoL perception (*e.g.*, restorations, extractions).

## Demographics and malocclusion types:

Demographic data, including age, gender and socioeconomic status (low, middle, high), were recorded for all participants. Malocclusion types were classified according to the Angle's Classification system:

[1] Class I: Normal bite alignment.

[2] Class II: Overbite or retrognathism.

[3] Class III: Underbite or prognathism.

This demographic data was used to analyze any potential correlations between patient characteristics and OHRQoL outcomes.

## Orthodontic treatment modalities:

[1] Ceramic Braces Group: Patients in this group received fixed ceramic braces, typically consisting of tooth-colored brackets and archwires. Treatment was carried out by experienced orthodontists, with regular adjustments every 4-6 weeks.

[2] Aligners Group: Patients in this group were treated with clear plastic aligners (*e.g.*, Invisalign), with a customized treatment plan developed based on initial impressions and digital scanning. Aligners were typically worn for 20-22 hours per day, with a new set of aligners provided every 2 weeks.

## OHRQoL assessment tool:

The Oral Health-Related Quality of Life (OHRQoL) was assessed using a 10-item questionnaire specifically designed to capture various aspects of the patient's life affected by orthodontic treatment. The questionnaire was based on validated OHRQoL dimensions and covered areas such as (Annexure 1, Table 1):

[1] Aesthetic Concerns - Satisfaction with the appearance of the teeth.

[2] Discomfort - Frequency and severity of pain or irritation.

[3] Functional Limitations - Impact of treatment on eating, speaking and other daily activities.

[4] Social and Psychological Impact - Impact on self-confidence, social interactions and psychological well-being.

[5] Treatment Satisfaction - Overall satisfaction with treatment.

Each question was scored on a 5-point Likert scale, where:

[1] 1 = Strongly Disagree/Very Unsatisfied/Very Poor

[2] 2 = Disagree/Unsatisfied/Poor

[3] 3 = Neutral/Neither Satisfied nor Dissatisfied/Fair

[4] 4 = Agree/Satisfied/Good

[5] 5 = Strongly Agree/Very Satisfied/Very Good

## Data collection:

Data collection took place at baseline (before treatment) and at a follow-up stage after a minimum of 6 months of orthodontic treatment. Participants completed the OHRQoL questionnaire, which was administered by trained researchers to ensure consistency in the data collection process. The questionnaires were self-administered, but researchers were available for clarification if necessary.

## Statistical analysis:

The collected data were analyzed using both descriptive and inferential statistical methods. Descriptive statistics, including mean and standard deviation, were utilized to summarize the demographic characteristics of the participants and their oral health-related quality of life (OHRQoL) scores. Inferential statistics, such as independent t-tests and Chi-square tests, were employed to compare the OHRQoL scores between the ceramic braces and aligner groups. To further explore the factors influencing OHRQoL outcomes, multiple regression analysis was conducted, examining the influence of demographic factors and malocclusion types. A p-value of less than 0.05 was considered statistically significant for all tests. Ethical approval for the study was obtained from the institutional review board (IRB) of the respective institution and informed consent was acquired from all participants before their inclusion in the study. Despite these strengths, the study had some limitations. The cross-sectional design restricted the ability to draw causal conclusions and the fixed sample size was based on a specific range of malocclusions. Therefore, future longitudinal studies with larger and more diverse populations are recommended to ensure broader applicability of the findings.

## Annexure 1- Questionnaire:

[1] How satisfied are you with the appearance of your teeth before and after treatment?

(1 = Very Unsatisfied, 5 = Very Satisfied)

[2] Have you experienced any discomfort or pain during treatment?

(1 = No, 5 = Yes, Very Severe)

[3] How much has your treatment affected your ability to eat or speak?

(1 = Not at all, 5 = Very Much)

[4] Do you feel self-conscious about your dental appearance during treatment?

(1 = Not at all, 5 = Very Self-Conscious)

[5] Has your treatment affected your social interactions (*e.g.*, avoiding photos, less smiling)?

(1 = No, 5 = Yes, A lot)

[6] Do you feel more confident about your smile after starting treatment?

(1 = No Change, 5 = Significant Improvement)

[7] How often do you have to adjust or replace any parts of your braces/aligners?

(1 = Never, 5 = Very Frequently)

[8] How would you rate the overall impact of the orthodontic treatment on your quality of life?

(1 = Very Negative, 5 = Very Positive)

[9] Have you experienced any issues such as irritation or sores in the mouth due to treatment?

(1 = No, 5 = Yes, Very Severe)

[10] How would you rate your overall satisfaction with the treatment you are receiving?

(1 = Very Dissatisfied, 5 = Very Satisfied)

## Results:

The first group consisted of patients undergoing treatment with ceramic braces. The age range of participants in this group was 18-35 years. In terms of gender distribution, 60% of the participants were female, while 40% were male. Malocclusion types in this group were as follows: 50% of patients had Class I malocclusion, 30% had Class II and 20% had Class III malocclusion. Regarding socioeconomic status, the majority of participants in this group belonged to the middle to upper-middle class (Table 2). The second group included participants undergoing treatment with aligners. The age range in this group was 18-40 years, with a gender distribution of 55% female and 45% male. In terms of malocclusion types, 40% of the participants had Class I malocclusion, 40% had Class II and 20% had Class III. The socioeconomic status of the participants was largely in the upper-middle-class category (Table 3). A higher percentage of patients in the aligners group (100%) were from an upper-middle-class background, compared to 85% in the ceramic braces group (p=0.015). This difference is statistically significant, potentially affecting treatment choice and perception of treatment outcomes. The aligners group reported a higher percentage of patients with a balanced diet (85%) compared to the ceramic braces group (75%). However, this difference was not statistically significant (p=0.09), indicating that both groups maintain relatively similar nutrition types. A larger proportion of patients in the aligners group (75%) maintained good oral hygiene compared to the ceramic braces group (65%). However, the difference was not statistically significant (p=0.12), indicating that both treatment groups are relatively similar in terms of hygiene maintenance (Table 4). The OHRQoL analysis for both the Ceramic Braces and Aligners groups reveals distinct differences in patient experiences across various aspects of oral health-related quality of life. The aligners group generally reported higher satisfaction in terms of aesthetic appearance, with a mean score of 4.00, compared to 3.75 for the ceramic braces group. This suggests that aligners are perceived as more aesthetically pleasing due to their discreet nature. In terms of discomfort, the aligners group again outperformed the ceramic braces group, with a mean score of 4.00 versus 3.45, indicating that patients using aligners experienced less discomfort during treatment. Both groups showed similar scores for social interaction, with the aligners group slightly ahead at 4.02 compared to 3.70 in the ceramic braces group, indicating a slightly lesser impact on social confidence for aligner patients. When assessing pain or sensitivity, the ceramic braces group had a higher mean score of 3.40, suggesting more frequent or intense discomfort, while the aligners group reported a mean score of 3.75. The aligners group also showed less concern about aesthetic visibility in public (mean score 4.18) compared to the ceramic braces group (mean score 4.02). Overall, the aligners group reported higher satisfaction in most aspects of OHRQoL, particularly in aesthetics, comfort and public perception, indicating a more favorable experience. However, despite these differences, both groups showed similar responses regarding treatment duration, oral hygiene maintenance and psychological impact, emphasizing that both treatment options have a relatively comparable effect on these factors (Table 5) and (Table 6).

## Aesthetic appearance (Q1):

The aligners group reported significantly higher satisfaction with aesthetic appearance (mean score = 4.00) compared to the ceramic braces group (mean score = 3.75). This suggests that patients using aligners felt more confident about their treatment's cosmetic outcome.

## Discomfort (Q2):

Both groups reported some level of discomfort, but the aligners group experienced less discomfort overall (mean score = 4.00) compared to the ceramic braces group (mean score = 3.45).

## Impact on functionality (Q3):

The impact on functionality was slightly higher in the ceramic braces group (mean score = 3.80) compared to the aligners group (mean score = 4.00), indicating that aligners may provide better functional comfort.

## Social interaction (Q4):

Both groups were relatively similar in terms of the impact of orthodontic treatment on social interactions, with the aligners group (mean score = 4.02) slightly outperforming the ceramic braces group (mean score = 3.70).

## Pain or sensitivity (Q5):

Pain and sensitivity were higher in the ceramic braces group (mean score = 3.40) compared to the aligners group (mean score = 3.75), suggesting that ceramic braces might cause more discomfort in terms of pain.

## Aesthetic concerns in public (Q6):

The aligners group felt significantly less concerned about aesthetic visibility in public (mean score = 4.18) than the ceramic braces group (mean score = 4.02).

## Psychological impact (Q7):

The psychological impact was higher in the aligners group (mean score = 4.12) compared to the ceramic braces group (mean score = 4.00), suggesting that aligners might have a slightly more positive psychological impact due to their less visible nature.

## Treatment duration (Q8):

Both groups had similar levels of concern regarding treatment duration, with the aligners group (mean score = 3.92) showing slightly more satisfaction compared to the ceramic braces group (mean score = 3.50).

## Oral hygiene maintenance (Q9):

The aligners group reported better oral hygiene maintenance (mean score = 3.80) compared to the ceramic braces group (mean score = 3.70), which may be attributed to the ease of cleaning aligners.

## Overall satisfaction (Q10):

Overall satisfaction was higher in the aligners group (mean score = 4.10) compared to the ceramic braces group (mean score = 3.92), further supporting the aligners group's higher perceived OHRQoL.

The mean total OHRQoL score for the aligners group (37) was higher compared to the ceramic braces group (34). However, this difference was not statistically significant (p=0.09). This analysis highlights that aligners may offer superior OHRQoL outcomes, particularly in terms of aesthetics, comfort and social interactions. However, further studies with larger sample sizes are needed to confirm these findings (Table 7).

## Discussion:

Orthodontic treatment aims to correct malocclusion, improve dental aesthetics and restore optimal function by applying controlled forces to reposition teeth within the alveolar bone. The two prominent treatment modalities discussed in this study are ceramic braces and clear aligners [17, 18-19]. Ceramic braces, functioning through brackets and archwires, apply continuous mechanical forces to guide tooth movement and are favoured for their tooth-colored aesthetic appeal. They follow the traditional fixed appliance mechanism of action, where the arch wire generates pressure transmitted through the brackets to shift teeth [20, 21- 22]. In contrast, clear aligners (*e.g.*, Invisalign) utilize a series of removable, custom-fabricated, transparent trays that apply controlled, sequential forces to achieve desired tooth movements. Each set of aligners is worn for a specified duration, typically 1-2 weeks and gradually guiding teeth into alignment. Aligners work on the principle of programmed tooth movement, wherein certain teeth are activated per aligner set based on digital treatment planning [23, 24-25]. While both methods aim to achieve similar end goals, their mechanisms, aesthetics, hygiene convenience and patient experience vary, influencing treatment acceptance and perceived oral health-related quality of life (OHRQoL). This study assessed and compared the oral health-related quality of life (OHRQoL) among adult patients undergoing orthodontic treatment with either ceramic braces or clear aligners. Based on a structured 10-item questionnaire evaluating dimensions like aesthetics, discomfort, functionality, psychological impact and overall satisfaction, the findings revealed a trend favouring aligners over ceramic braces in most quality-of-life domains. The mean total OHRQoL score for the aligners group was 37, while the ceramic braces group scored 34, indicating better overall satisfaction among aligner users. Although this numerical difference was not statistically significant (p=0.09), it is clinically relevant, as even small differences in patient-perceived quality of life can impact treatment compliance and acceptance.

Patients using aligners reported a significantly higher mean score for aesthetic satisfaction (Q1 = 4.00) compared to those with ceramic braces (Q1 = 3.75). This aligns with findings from Pithon *et al.* (2014) [26] who emphasized those aligners are more accepted by adult patients due to their invisibility and discreet nature, which minimizes social anxiety during treatment. Similarly, concerns about aesthetic visibility in public (Q6) were lower in the aligners group (mean = 4.18) compared to ceramic braces (mean = 4.02), reinforcing the cosmetic appeal of aligners. Aligner users reported less discomfort (Q2 = 4.00) than ceramic braces users (Q2 = 3.45). This is in agreement with Rosvall *et al.* (2009) [27], who found that fixed appliances, even ceramic ones, are associated with more soft tissue irritation and pressure pain due to bracket-bonded surfaces. Aligners, being smooth and removable, lead to fewer ulcerations and pressure points, likely accounting for the higher comfort ratings. Similarly, pain or sensitivity (Q5) scores were also better for aligners (3.75 vs. 3.40 for ceramic braces), suggesting less tissue trauma and inflammation. Aligners showed a marginal advantage in terms of functional limitations (Q3 = 4.00) versus ceramic braces (Q3 = 3.80). Aligners can be removed during eating and oral hygiene practices, which may explain their higher functional ratings. Social impact scores (Q4) were nearly similar, but slightly higher in aligners (4.02) than in ceramic braces (3.70), suggesting that aligners may contribute to better self-esteem and confidence in social settings. This is supported by Shalish *et al.* (2012) [28], who concluded that adults using aligners demonstrated better psychological and social well-being throughout orthodontic treatment. In terms of psychological comfort (Q7), aligners again scored slightly better (4.12 vs. 4.00). The perception of having control over treatment, discreet appearance and fewer dietary restrictions likely contributed to this outcome. The overall satisfaction (Q10) was also higher in the aligners group (4.10 vs. 3.92), showing that aligner patients felt more positively about their orthodontic journey. Although oral hygiene maintenance (Q9) scores were slightly better in the aligners group (3.80) compared to ceramic braces (3.70), the difference was minimal. The ease of removing aligners for brushing and flossing may provide an advantage, aligning with findings from Spirito *et al.* (2023) [29] that fixed appliances often lead to plaque accumulation if hygiene is not meticulously maintained. When comparing lifestyle factors, nutrition and hygiene patterns were relatively similar between groups. However, 85% of aligner patients reported consuming a balanced diet compared to 75% in the ceramic braces group (p=0.09) and 75% of aligner users maintained good hygiene compared to 65% in the ceramic group (p=0.12). These factors, though not statistically significant, might contribute to enhanced patient comfort and fewer oral complications in the aligner group.

A significant finding was the socioeconomic distribution: 100% of aligner patients were from an upper-middle-class background compared to 85% in the ceramic braces group (p=0.015). This is consistent with literature indicating that the cost of aligner therapy can limit its accessibility to higher-income populations [30]. The main purpose of orthodontic procedure is to treat malocclusion. Zamora-Martínez *et al.* found, significant decrease in quality of life during orthodontic treatment compared to their pre-treatment condition [31]. Affordability and awareness may influence treatment choice, expectations and patient satisfaction.In summary, while both ceramic braces and aligners improved oral health-related quality of life in adult orthodontic patients, aligners showed higher mean scores in nearly all questionnaire domains, including aesthetics, comfort, pain, psychological impact and overall satisfaction. These findings support existing literature favouring aligners for their discreet appearance and improved patient-reported outcomes. However, the lack of statistically significant differences (except for socioeconomic status) suggests that both treatment modalities remain effective and acceptable. Larger, multicenter and longitudinal studies are recommended to further validate these results and explore long-term effects on OHRQoL. The study was limited by its small sample size and single-centre design, which may affect the generalizability of the findings. Future research, should focus on long-term, multicentre trials with diverse populations to better understand the sustained impact of different orthodontic treatments on OHRQoL.

## Conclusion:

Data shows valuable insights into the OHRQoL of adults undergoing orthodontic treatment with ceramic braces versus aligners. Aligners appear to offer a more comfortable and aesthetically pleasing option, though both treatments are effective in improving oral health-related quality of life.

## Figures and Tables

**Table 1:** Scoring for answer options

**Question No.**	**Answer Options**	**Score Range**
1	Very Unsatisfied to Very Satisfied	5-Jan
2	No to Very Severe	5-Jan
3	Not at all to Very Much	5-Jan
4	Not at all to Very Self-Conscious	5-Jan
5	No to Yes, A lot	5-Jan
6	No Change to Significant Improvement	5-Jan
7	Never to Very Frequently	5-Jan
8	Very Negative to Very Positive	5-Jan
9	No to Yes, Very Severe	5-Jan
10	Very Dissatisfied to Very Satisfied	5-Jan

**Table 2:** Ceramic braces group

**Parameter**	**Ceramic Braces Group (n=100)**
Age Range	18-35 years
Gender	60% Female, 40% Male
Malocclusion Types	Class I: 50%, Class II: 30%, Class III: 20%
Socioeconomic Status	Middle to Upper-middle class
Nutrition Type	Balanced Diet: 75%, High-Sugar Diet: 25%
Hygiene Maintenance	Good: 65%, Moderate: 30%, Poor: 5%

**Table 3:** Aligners group

**Parameter**	**Aligners Group (n=100)**
Age Range	18-40 years
Gender	55% Female, 45% Male
Malocclusion Types	Class I: 40%, Class II: 40%, Class III: 20%
Socioeconomic Status	Upper-middle class
Nutrition Type	Balanced Diet: 85%, High-Sugar Diet: 15%
Hygiene Maintenance	Good: 75%, Moderate: 20%, Poor: 5%

**Table 4:** Comparative analysis for socioeconomic status, nutrition, hygiene maintenance

**Factor**	**Ceramic Braces Group (n=100)**	**Aligners Group (n=100)**	**p-value**
Socioeconomic Status	Middle-Upper Class: 85%	Upper-Middle Class: 100%	0.015
Nutrition Type	Balanced Diet: 75%, High-Sugar Diet: 25%	Balanced Diet: 85%, High-Sugar Diet: 15%	0.09
Hygiene Maintenance	Good: 65%, Moderate: 30%, Poor: 5%	Good: 75%, Moderate: 20%, Poor: 5%	0.12

**Table 5:** Ceramic Braces Group: Question-wise Breakdown

**Question**	**1**	**2**	**3**	**4**	**5**	**Mean Score**
Q1: Aesthetic Appearance	5%	10%	25%	35%	25%	3.75
Q2: Discomfort	10%	15%	25%	30%	20%	3.45
Q3: Impact on Functionality	3%	10%	25%	35%	27%	3.8
Q4: Social Interaction	8%	10%	20%	35%	27%	3.7
Q5: Pain or Sensitivity	12%	18%	25%	30%	15%	3.4
Q6: Aesthetic Concerns in Public	7%	10%	20%	30%	33%	4.02
Q7: Psychological Impact	5%	8%	25%	30%	32%	4
Q8: Treatment Duration	10%	15%	30%	25%	20%	3.5
Q9: Oral Hygiene Maintenance	3%	12%	30%	35%	20%	3.7
Q10: Overall Satisfaction	6%	10%	25%	30%	29%	3.92

**Table 6:** Aligners Group: Question-wise Breakdown

**Question**	**1**	**2**	**3**	**4**	**5**	**Mean Score**
Q1: Aesthetic Appearance	3%	7%	20%	35%	35%	4
Q2: Discomfort	5%	10%	20%	25%	40%	4
Q3: Impact on Functionality	4%	8%	20%	35%	33%	4
Q4: Social Interaction	3%	10%	25%	30%	32%	4.02
Q5: Pain or Sensitivity	8%	12%	25%	30%	25%	3.75
Q6: Aesthetic Concerns in Public	4%	8%	18%	30%	40%	4.18
Q7: Psychological Impact	3%	5%	22%	25%	45%	4.12
Q8: Treatment Duration	6%	10%	28%	30%	26%	3.92
Q9: Oral Hygiene Maintenance	2%	8%	30%	35%	25%	3.8
Q10: Overall Satisfaction	3%	5%	20%	35%	37%	4.1

**Table 7:** Total OHRQoL Scores

**Group**	**Mean Total OHRQoL Score**	**Standard Deviation**	**p-value**
Ceramic Braces Group	34	4.52	0.09
Aligners Group	37	4	N/A
